# Adaptive laboratory evolution and transcriptomic profiling reveal carbon–nitrogen metabolic reprogramming enabling aerobic co-fermentation of glucose and xylose in *Saccharomyces cerevisiae*

**DOI:** 10.1371/journal.pone.0341927

**Published:** 2026-01-30

**Authors:** Lina Maria López-deÁvila, Zulma Isabel Monsalve-Fonnegra, Héctor Alejandro Rodríguez-Cabal

**Affiliations:** 1 Grupo Biotransformación, Escuela de Microbiología, Universidad de Antioquia, Medellín, Colombia; 2 Grupo Agrobiotecnología, Facultad de Ciencias Exactas y Naturales, Universidad de Antioquia, Medellín, Colombia; 3 Grupo de Biotecnología Vegetal, Universidad Nacional de Colombia sede Medellín, Facultad de Ciencias Agrarias, Departamento de Ciencias Agronómicas, Medellín, Colombia; Osmania University, INDIA

## Abstract

The efficient conversion of lignocellulosic sugars into bioethanol is constrained by the inability of *Saccharomyces cerevisiae* to metabolize xylose and by its preference for glucose when both sugars are available. Although recombinant strains have been developed to improve xylose utilization, further optimization is needed to achieve robust co-fermentation performance. In this study, three parental strains were used: a wild-type *S. cerevisiae* strain (GF16), a genetically engineered *S. cerevisiae* strain capable of metabolizing xylose (TMB3001), and a reference strain of *Scheffersomyces stipitis* (ATCC 58376). From these, we obtained an evolved *S. cerevisiae* strain (F2C7A) through a combination of UV mutagenesis, protoplast fusion, and adaptive laboratory evolution. In synthetic medium containing only xylose, F2C7A consumed 87.9% of the sugar after 72 h, compared with only 52.3% by its parental hybrid strain, although, its biomass yield was lower (0.20 g/g vs. 0.35 g/g). Under mixed-sugar conditions, F2C7A consumed all available glucose and 33% of xylose within 48 h, producing ethanol at 0.45 g/g yield with minimal xylitol accumulation. In culture medium containing only xylose, it reached a biomass yield of 0.86 g/g and a xylitol yield of 0.11 g/g. Transcriptomic analysis revealed strong induction of *XYL1*, *XYL2*, tricarboxylic acid cycle genes, and oxidative phosphorylation components under xylose, consistent with a respiratory phenotype. Mixed-sugar cultures displayed a respirofermentative profile and reduced xylitol formation, suggesting improved redox balance in the presence of glucose. Several nonspecific sugar transporters (*HXT8*, *HGT1*, *STL1*) were overexpressed under xylose, indicating potential compensatory uptake mechanisms. Changes in nitrogen metabolism included upregulation of *GLT1* and repression of *GDH1*, suggesting a shift toward NADH-dependent glutamate synthesis. These findings demonstrate that combining classical and evolutionary strategies can enhance xylose metabolism in *S. cerevisiae*, providing a foundation for further improvement of strains intended for lignocellulosic bioethanol production.

## Introduction

The increasing global demand for sustainable energy sources has intensified research into lignocellulosic biomass as a renewable feedstock for bioethanol production [1,2]. Lignocellulosic hydrolysates are rich in hexoses, such as glucose, and pentoses, particularly xylose, the latter comprising up to 30% of total fermentable sugars [3]. Efficient co-fermentation of glucose and xylose is thus essential for achieving economically viable second-generation bioethanol production. However, *Saccharomyces cerevisiae*, the organism most widely used in industrial fermentation, naturally lacks the ability to ferment xylose and preferentially consumes glucose when both sugars are present [4].

While other yeast species such as *Scheffersomyces stipitis*, *Pachysolen tannophilus*, and *Candida shehatae* can assimilate xylose, their low ethanol productivity and limited stress tolerance render them unsuitable for industrial-scale applications [5]. Metabolic engineering has enabled *S. cerevisiae* to assimilate xylose by introducing heterologous pathways, such as the xylose reductase (XR) and xylitol dehydrogenase (XDH) pathway from *S. stipitis* or the isomerase pathway (XI) [6–8]. Although these engineered strains can metabolize xylose under laboratory conditions, their performance remains limited by low xylose consumption rates, reduced ethanol yields, and sensitivity to inhibitors present in lignocellulosic hydrolysates [9]. Additionally, regulatory and metabolic constraints, including glucose repression and redox imbalance, further restrict efficient xylose utilization in engineered yeasts [10].

To overcome these limitations, complementary approaches such as genome shuffling and adaptive laboratory evolution (ALE) have been applied. Genome shuffling accelerates strain improvement by combining beneficial alleles from different parental strains through recursive protoplast fusion [11,12], while ALE enables the accumulation of adaptive mutations under defined and prolonged selection pressures. Together, these approaches complement rational metabolic engineering by optimizing complex regulatory networks that are difficult to fine-tune by design. Previous studies have shown that both genome shuffling and ALE can enhance stress tolerance, substrate utilization, and ethanol productivity in yeast [13–15].

Recently, hybrid strain improvement approaches that integrate both natural and synthetic diversity have recently gained increasing attention. Interspecific hybrids between *S. cerevisiae* and *S. stipitis* have shown enhanced ethanol production under multiple stress conditions [16] and improved fermentative capacity in strains with high evolutionary potential [17]. Wang et al. (2022) reported that the hybrid *S. cerevisiae* strain E-158 produced ethanol concentrations 10.14%–81.02% higher than those obtained with its parental strain under high ethanol, high temperature, and osmotic stress conditions [16]. Similarly, Pérez et al. (2023) described interspecific *S. cerevisiae* hybrids with improved aromatic profiles that reduced ethanol content and increased organic acid production [18]. These results highlight the potential of evolutionary engineering to broaden the physiological capacity of both recombinant and industrial strains.

In this study, one of the parental strains used for hybrid generation (*S. cerevisiae* TMB3001) was previously engineered to metabolize xylose via the *S. stipitis* XR/XDH pathway [19]. Here, our goal, was to enhance and diversify xylose metabolism through non-GMO strategies. By combining UV mutagenesis, genome shuffling, and ALE, we aimed to generate adaptive variants with improved redox balance, sugar co-utilization, and metabolic robustness under aerobic conditions.

Despite notable advances in rational strain engineering, there remains a critical gap in developing *S. cerevisiae* strains capable of robust xylose assimilation through the integration of natural diversity and evolutionary selection. While recombinant DNA strategies are essential to introduce heterologous xylose pathways, they require extensive metabolic rewiring and are limited by incomplete knowledge of global regulatory networks. In contrast, non-GMO approaches such as classical mutagenesis, genome shuffling, and ALE allow genome-wide adaptive improvements without direct genetic manipulation, enabling the emergence of complex traits—such as tolerance, efficient substrate utilization, and redox balance—that are often difficult to achieve through rational design alone. The novelty of this work lies in demonstrating how sequential application of genome shuffling and ALE can potentiate xylose metabolism in a hybrid background derived from both native and recombinant lineages. Specifically, this study (i) develops evolved *S. cerevisiae* hybrids capable of co-fermenting glucose and xylose under aerobic conditions, (ii) characterizes their metabolic and transcriptomic adaptations, and (iii) identifies redox- and nitrogen-related regulatory mechanisms underlying improved co-fermentation performance. These findings provide new insights into adaptive network remodeling in yeast and establish a framework for designing robust, non-GMO yeast platforms for lignocellulosic bioethanol production.

## Materials and methods

### Strains and media

*S. cerevisiae* TMB3001 [19], *S. cerevisiae* GF16 (isolated from Isabella grapes) and *S. stipitis* ATCC 58376 (NRRL Y-7124) [20], were used as parental strains for strain development. All strains were maintained on YPD medium (1% yeast extract, 2% peptone, 2% glucose) at 35 °C. Fermentation assays were conducted using synthetic YNB medium (6.7 g/L yeast nitrogen base without amino acids, Sigma-Aldrich) supplemented with 2% D-xylose and 0.1% D-glucose (YNBX). Routine cultures were grown on YPX medium (1% yeast extract, 2% peptone, 2% xylose). Long-term storage was performed in 30% glycerol at –80 °C.

### UV mutagenesis

UV mutagenesis followed the protocol of Winston (2008) [21] for *S. cerevisiae*. Overnight YPD cultures of parental strains were harvested, washed twice with sterile 0.9% NaCl, and adjusted to 2 × 10⁸ cells/mL. Aliquots were plated on YPX agar and exposed to UV irradiation (254 nm, 1500 μJ/cm²) for 60–480 s. A 300 s exposure, yielding ~30% survival, was used for mutagenesis. To stop photoreactions, plates were incubated in the dark at 35 °C for 96 h. Colonies were replicated on YPX agar, incubated at 35 °C for 48 h, and mutants capable of xylose growth were confirmed by re-growth on YPD and YPX.

### Genome shuffling

Mutants with the highest xylose consumption on YPX were selected for protoplast fusion, adapted from Hou et al. (2012) [22] and Yin et al. (2016) [23]. YPD-grown cells were incubated in phosphate buffer (0.096 M KH₂PO₄/0.05 M Na₂HPO₄, pH 5.6) with 0.1% EDTA and 0.1% 2-mercaptoethanol at 30 °C for 30 min, then washed with a sorbitol-based buffer (0.9 M sorbitol, 0.1 M EDTA, 0.05 M DTT, pH 7.5) and treated with lyticase (625 U/g cells) at 30 °C for 30 min. Protoplast formation was confirmed microscopically.

Equal volumes of protoplast suspensions (10⁶ protoplasts/mL) from each mutant were mixed and split into two fractions. One fraction was UV-inactivated (254 nm, 30 cm distance, 30 min), and the other was heat-inactivated at 60 °C for 30 min [24,25]. Fractions were combined in a 1:1 ratio, centrifuged, resuspended in fusion buffer (phosphate buffer with 40% PEG 6000 and 0.01 M CaCl₂), incubated at 30 °C for 20 min, then washed with sterile 0.9% NaCl. The resulting suspension was diluted and spread onto protoplast regeneration plate (YPX agar with 0.6 M KCl and 0.025 M CaCl₂), and cultured at 30 °C for 4 d. Colonies recovered from these plates were considered F1 hybrids. Biomass formation and xylose consumption were determined, and the strains showing the highest xylose consumption were selected for further recombination. Selected F1 strains were fused among themselves to maximize genome shuffling, and the process was repeated for three consecutive cycles. The best-performing hybrids were maintained on YPX plates for subsequent analysis.

### Adaptive laboratory evolution (ALE)

Two hybrid strains (F2C7 and F3C12) were selected for ALE. Single colonies were inoculated into 250 mL Erlenmeyer flasks with 100 mL YNBX medium (20 g/L xylose) and incubated at 35 °C, 120 rpm until complete xylose consumption. For subsequent cycles, cultures were transferred to fresh YNBX at an initial OD₆₀₀ of 0.1. At each transfer, samples were plated on YNBX agar, and individual colonies were isolated and stored in 30% glycerol at –80 °C.

### Shake flask fermentation

Fermentation assays were performed in YNBX under aerobic and anaerobic conditions. Aerobic cultures were incubated in 250 mL flasks with 50 mL medium and cotton plugs. Anaerobic conditions were established using 150 mL medium in tightly sealed flasks with CO₂ traps. All cultures were inoculated at OD₆₀₀ = 0.1 from overnight precultures, incubated at 35 °C, 120 rpm for 72 h. Samples were collected every 24 h and stored at –20 °C.

### Analytical methods

Cell growth was monitored by measuring OD₆₀₀ with a Genesys™ 10S UV-Vis spectrophotometer (Thermo Scientific, USA). To determine cell dry weight (CDW), *S. cerevisiae* was cultivated in YPD medium until stationary phase and centrifuged at 5,000 × g for 10 min. The cell pellet was washed twice with sterile 0.9% NaCl and resuspended in 10 mL of the same solution. Serial 1:2 dilutions were prepared and filtered through pre-dried and pre-weighed membranes (0.45 µm). The membranes were dried at 105 °C until constant weight. OD₆₀₀ of the dilutions was measured, and a calibration curve was constructed by correlating OD values with CDW measurements. Glucose, xylose, xylitol, glycerol, and ethanol concentrations were determined by HPLC (Agilent Technologies, USA) with an Aminex HPX-87H column (Bio-Rad, USA), using 0.005% H₂SO₄ as mobile phase at a flow rate of 0.6 mL/min and 65 °C. Samples were centrifuged and filtered through 0.22 µm nylon membranes prior to analysis.

### RNA extraction, sequencing, and transcriptomic analysis

Strain F2C7A was grown in YPD, YPX, and YPXD (1% glucose + 1% xylose) at 35 °C and 120 rpm. Samples were taken after 24 h from triplicate cultures. Total RNA was extracted using the GeneJet RNA Purification Kit (Thermo Scientific, USA), according to the manufacturer’s instructions. RNA quality and concentration were assessed via NanoDrop 2000 (Thermo Fisher, USA) and a 5400 Fragment Analyzer System (Agilent Technologies, USA).

Library preparation and sequencing were performed by Novogene (CA, USA). mRNA was isolated using poly-A magnetic beads, and libraries were sequenced on a NovaSeq PE150 platform. Clean reads were aligned to the *S. cerevisiae* S288C reference genome (GCA_000146045.2) using HISAT2 v2.0.5. Gene expression was quantified as fragments per kilobase of exon per million reads mapped (FPKM). Differential gene expression across glucose (GLU), xylose (XIL), and mixed sugar (XILGLU) conditions were analyzed with DESeq2 v1.16.1. Genes with adjusted *p* < 0.05 and log₂ fold change ≥ 1 or ≤ −1 were considered differentially expressed. Gene Ontology (GO) and KEGG pathway enrichment analyses were performed using the ClusterProfiler R package. Enriched terms were defined by adjusted p-value < 0.05.

### Statistical analysis

All data are presented as mean ± standard deviation (SD) from three independent biological replicates. Statistical differences among groups were evaluated by one-way ANOVA followed by Tukey’s HSD test, with significance set at *p* < 0.05. For pairwise comparisons, Student’s *t*-*t*est was applied. All analyses were performed using GraphPad Prism 10 (GraphPad Software, San Diego, CA, USA).

## Results

### Enhancement of genetic diversity and selection of xylose-consuming hybrids

To obtain an evolved yeast strain with improved xylose consumption, ethanol production, and stress tolerance, three parental strains were initially selected: *Saccharomyces cerevisiae* TMB3001, a recombinant strain carrying the *XYL1* and *XYL2* genes; *S. cerevisiae* GF16, a native osmotolerant isolate; and *S. stipitis* ATCC 58376, a natural pentose-fermenting yeast.

**Fig 1:**
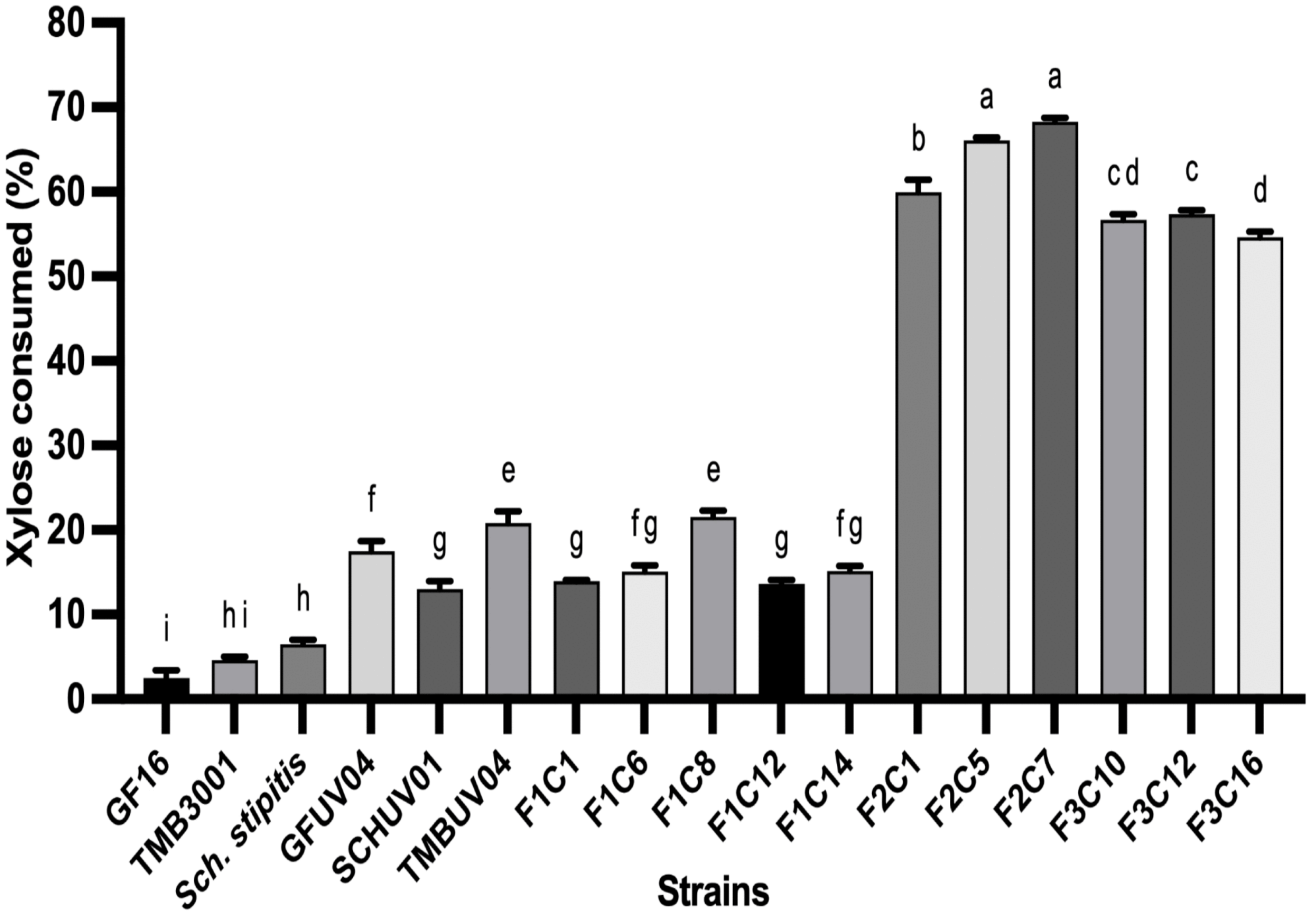
Improvement of xylose utilization in UV-mutant and hybrid strains. (A) Xylose consumption (%) by parental, UV-mutated and hybrid strains obtained after three rounds of genome shuffling (F1, F2 and F3) after 48 hours of cultivation in YNB medium containing 20 g/L xylose and 1 g/L glucose under aerobic conditions. Bars represent the mean ± SD of three independent biological replicates. Different letters above the bars indicate statistically significant differences between strains (one-way ANOVA followed by Tukey’s HSD test, *p* < 0.05).

These strains were subjected to UV mutagenesis and selected on xylose-containing medium, yielding a mutant library of 21 colonies. In YPX medium, mutants GFUV04, TMBUV04, and SCHUV01 exhibited 9-fold, 4-fold, and 2-fold increases in xylose consumption, respectively, compared to their parental strains (*p* < 0.01, one-way ANOVA; Fig 1). The GFUV04 mutant showed a 32% decrease in biomass production, whereas SCHUV01 achieved a twofold increase in biomass (p < 0.01).

The selected mutants were used in three successive rounds of protoplast fusion. In the first round (F1), 14 fusants were obtained from which five (F1C1, F1C6, F1C8, F1C12, and F1C14) were selected. In the second round, three F2 hybrids (F2C1, F2C5, and F2C7) demonstrated xylose consumption ranging from 61–69% after 48 hours, with F2C7 exhibiting the best performance (12.1 ± 1.3 g/L). A third round of genome shuffling did not result in further improvement (Fig 1).

### Adaptive laboratory evolution improves xylose utilization and biomass formation

**Fig 2:**
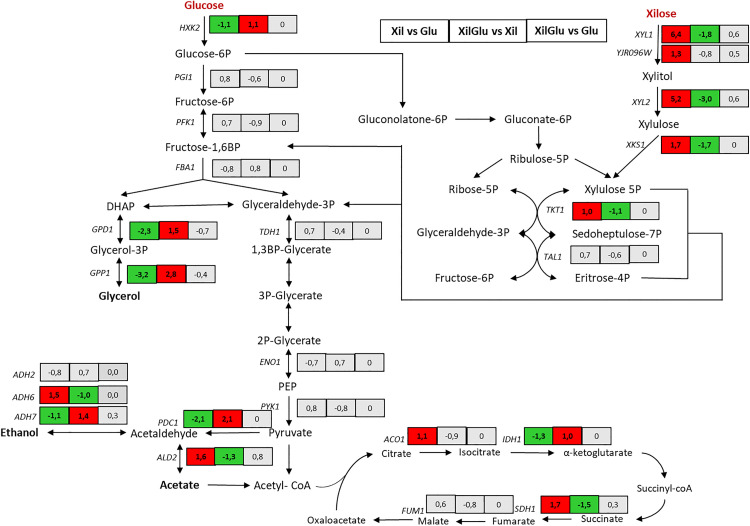
Differential expression of central carbon metabolism genes in evolved strain F2C7A under mixed-sugar and single-sugar conditions.

Hybrid strains F2C7 and F3C12 were selected for adaptive laboratory evolution (ALE) in YNBX medium. During the first ALE cycle, both strains exhibited an extended lag phase (72 h for F2C7 and 96 h for F3C12; Fig 2A and 2C) and slow growth. In the second cycle, the evolved strains exhibited significantly shorter lag phase and increased specific growth rates, reaching 0.06 ± 0.01 h ⁻ ¹ for F2C7 and 0.04 ± 0.01 h ⁻ ¹ for F3C12, corresponding a 3.0- and 1.4-fold (p < 0.05) increases compared to the growth rate observed in the first cycle. Consistently, xylose consumption was markedly enhanced (Fig 2B and 2D). En F2C7, 15.7 ± 3.2 g/L of the initial xylose (20 g/L) was consumed by the end of the first cycle (168h), whereas in the second cycle, almost complete xylose depletion was achieved within 120 h. In F3C12, xylose consumption increased by 27% in the second cycle. From these adapted populations, individual colonies were isolated and designated F2C7A and F3C12A; these evolved isolates were used in all further phenotypic and transcriptomic experiments. Together, the data indicate that ALE produced substantial improvements in specific xylose uptake rate and overall metabolic responsiveness.

**Fig 3:**
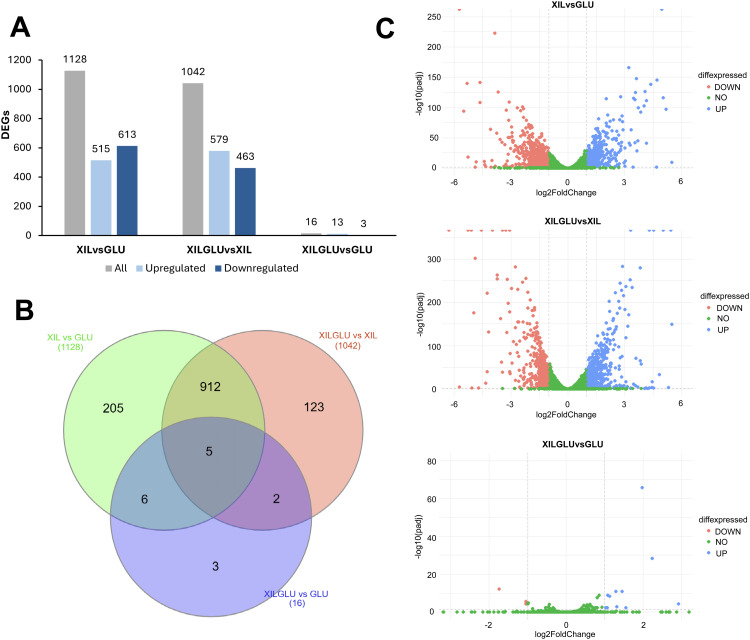
Transcriptomic analysis of differentially expressed genes (DEGs) under glucose, xylose, and mixed carbon sources. (A) Number of DEGs identified in pairwise comparisons between growth conditions: xylose vs. glucose (XIL vs. GLU), mixed xylose–glucose vs. xylose (XILGLU vs. XIL), and mixed xylose–glucose vs. glucose (XILGLU vs. GLU). (B) Venn diagram showing unique and shared DEGs among the three comparisons. (C) Volcano plot illustrating the distribution of DEGs according to log_2_ fold change (log_2_FC) and adjusted p-value in each comparison. Genes with log_2_FC ≥ 1 and adjusted p-value < 0.05 were considered differentially expressed.

To evaluate the phenotypic effect of ALE experiment on xylose metabolism, the evolved isolates F2C7A and F3C12A were cultivated, along with their parental hybrid strains, in fresh YNBX medium for 72 h. As shown in Fig 3B, both evolved strains exhibited significantly enhanced xylose utilization compared to hybrid strains. F2C7A consumed 87.9% of the available xylose, while its parental strain consumed only 52.3% under the same conditions. Similarly, F3C12A increased xylose consumption from 21.7% in the hybrid strain to 70.1% after evolution. This improvement in xylose consumption was accompanied by a significant increase in biomass formation. F3C12A reached a final CDW of 9.0 ± 0.8 g/L after 72 h, representing nearly a 5-fold increase compared to its non-evolved parental strain (Fig 3A). While F2C7A achieved a similar final biomass concentration to F2C7, its biomass yield was reduced (Y_Biomass_ of 0.20 g/g vs. 0.35 g/g).

Under both aerobic and anaerobic conditions, evolved strains F2C7A and F3C12A exhibited distinct metabolic profiles in YNBX medium (Table 1). Both strains showed enhanced growth under aerobic conditions. F2C7A reached a biomass concentration of 3.6 ± 0.2 g/L and consumed 87.2% of the available xylose, with a specific growth rate of 0.125 h ⁻ ¹ and a biomass yield of 0.20 g/g. In comparison, F3C12A achieved significantly higher biomass accumulation (9.4 ± 0.6 g/L) and a greater biomass yield (0.63 g/g), despite consuming slightly less xylose (74.9%). Under anaerobic conditions, both strains exhibited reduced growth. F2C7A produced 1.0 ± 0.1 g/L of biomass and consumed 60.4% of xylose, while F3C12A accumulated 2.3 ± 0.4 g/L of biomass with only 24.1% xylose consumption. Interestingly, F3C12A displayed a higher biomass yield under oxygen-limited conditions (0.47 g/g) compared to F2C7A (0.08 g/g). However, F2C7A exhibited the highest specific xylose consumption rate under anaerobic conditions (0.18 g/g h ⁻ ¹), suggesting increased fermentative activity.

**Table 1:** Kinetic parameters of evolved strains F2C7A and F3C12A grown in YNBX medium under aerobic and anaerobic conditions.

Strain	Oxygen Condition	Biomass (g/L)	Consumed xylose (%)	µ_max_	Y_Biomass_	r_Xylose_	Y_Ethanol_
F2C7A	Aerobic	3.55 ± 0.15	87.2	0.125	0.20	0.07	0.18
	Anaerobic	0.99 ± 0.12	60.4	0.059	0.08	0.18	0.07
F3C12A	Aerobic	9.43 ± 0.60	74.9	0.092	0.63	0.02	0.00
	Anaerobic	2.33 ± 0.41	24.1	0.090	0.47	0.03	0.00

Strains were cultivated in YNBX medium containing 20 g/L xylose and 1 g/L glucose. Parameters were calculated at 72 hours, when all cultures had reached the stationary phase. Parameters: final biomass (g/L); percentage of consumed xylose; µ_max_, volumetric growth rate (h ⁻ ¹); Y_Biomass_, biomass yield (g biomass/g xylose); r_Xylose_, xylose consumption rate (g/g h ⁻ ¹); (Y_Ethanol_, ethanol yield (g ethanol/g xylose).

Overall, F2C7A showed lower carbon flux from xylose toward biomass formation, particularly under aerobic conditions, where it had a lower biomass yield, but a higher xylose consumption rate (0.07 g/g h ⁻ ¹) compared to F3C12A (0.02 g/g h ⁻ ¹). These findings suggest that F2C7A may favor a more fermentative metabolism, while F3C12A channels more xylose carbon toward biomass production, particularly in the presence of oxygen.

### Respirofermentative metabolism and carbon flux distribution in media with single and mixed sugars

**Fig 4:**
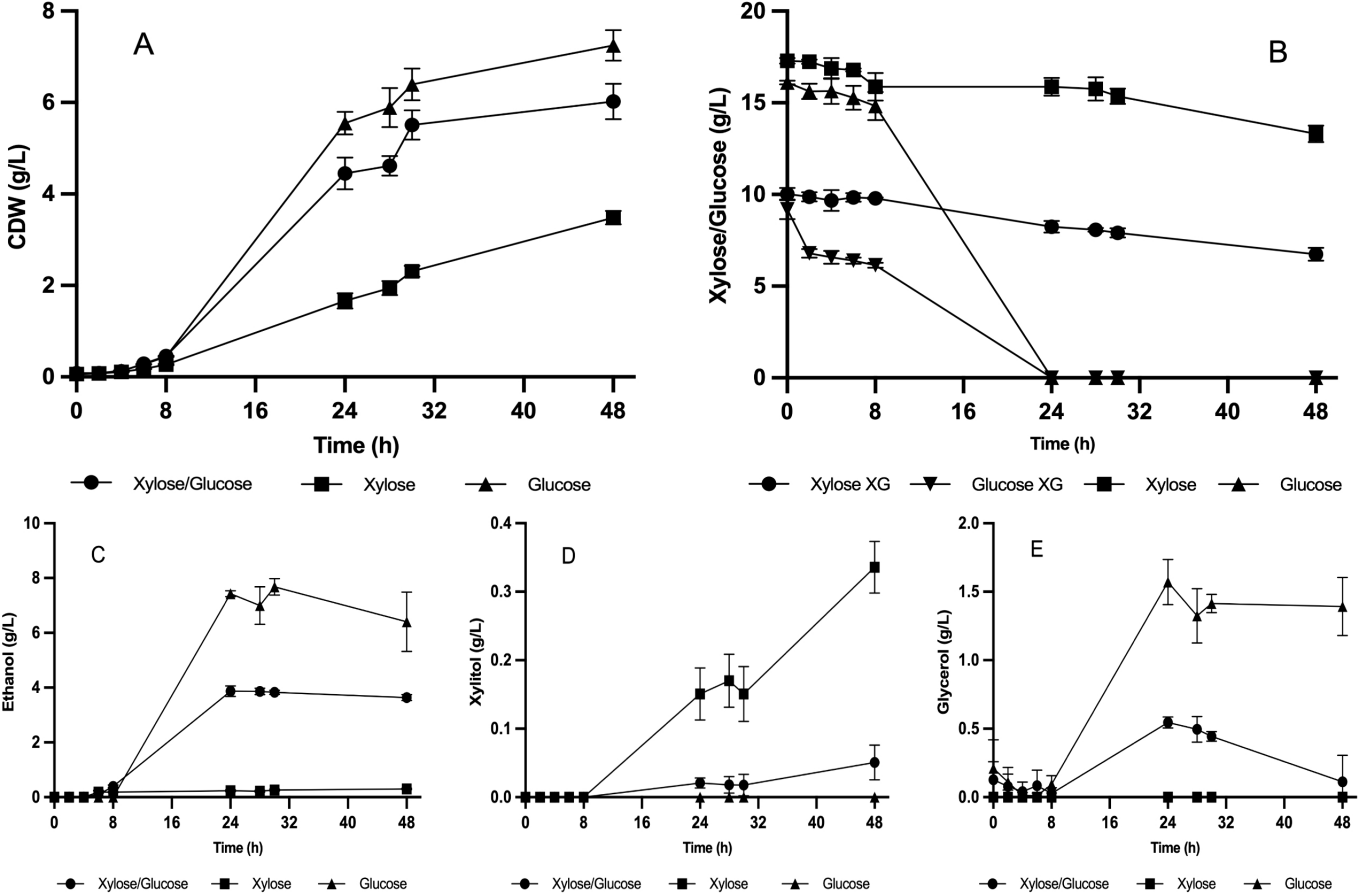
Fermentation profile of strain F2C7A in YP media supplemented with glucose and/or xylose. F2C7A was cultivated aerobically in YP medium containing 20 g/L of glucose (YPD), 20 g/l xylose (YPX), or both sugars (YPXD). (A) biomass formation, (B) Sugar consumption, (C) ethanol production, (D) xylitol accumulation and (E) glycerol production were monitored for 72 **h.** Values represent mean ± SD from three independent biological replicates.

To further explore the metabolic capacity of the evolved strain F2C7A, subsequent fermentations were conducted in YP medium supplemented with glucose (YPD), xylose (YPX), or a mixture of both sugars (YPXD). As shown in Fig 4, substrate consumption, growth, and metabolite production were strongly influenced by the carbon source. In YPD medium, glucose was rapidly consumed within the first 24 h, leading to the highest ethanol yield (0.46 g/g) and biomass production (7.3 ± 0.4 g/L), consistent with fermentative metabolism. In contrast, growth in YPX medium resulted in slower sugar consumption, with a specific xylose uptake rate of g/g h ⁻ ¹. Cultures in these complex medium revealed clear differences in sugar utilization compared with the minimal YNBX medium used previously. Since YPX contains yeast extract and peptone as additional sources of carbon and nitrogen, F2C7A exhibited robust growth, reaching biomass concentration of 3.48 ± 0.18 g/L. The presence of alternative carbon sources likely supported biomass. Ethanol production was minimal, indicating primarily respiratory metabolism. In the mixed YPXD medium, both sugars were consumed simultaneously. Glucose was completely depleted within the first 24 h, while 18% of the available xylose was also consumed during this time. This indicates that, although glucose was the preferred substrate, xylose assimilation was not fully repressed. Ethanol yield reached 0.51 g/g, suggesting a respirofermentative profile. These results indicate that the strain maintained glucose fermentation capacity even in the presence of xylose and under aerobic conditions.

Additionally, xylitol production was detected exclusively in YPX medium, reaching 0.44 ± 0.18 g/L, suggesting a redox imbalance during xylose metabolism under these conditions. In contrast, xylitol levels were markedly lower in the mixed YPXD medium (0.03 ± 0.01 g/L), indicating that the presence of glucose may help resolve the redox imbalance associated with xylose assimilation. Glycerol production remained very low under all tested conditions. These findings support the hypothesis that in the presence of both carbon sources, F2C7A exhibits a respirofermentative metabolism that balances redox cofactors more efficiently, favoring biomass and ethanol production while minimizing by-product accumulation.

### Transcriptomic response of F2C7A to xylose and glucose under aerobic conditions

**Fig 5:**
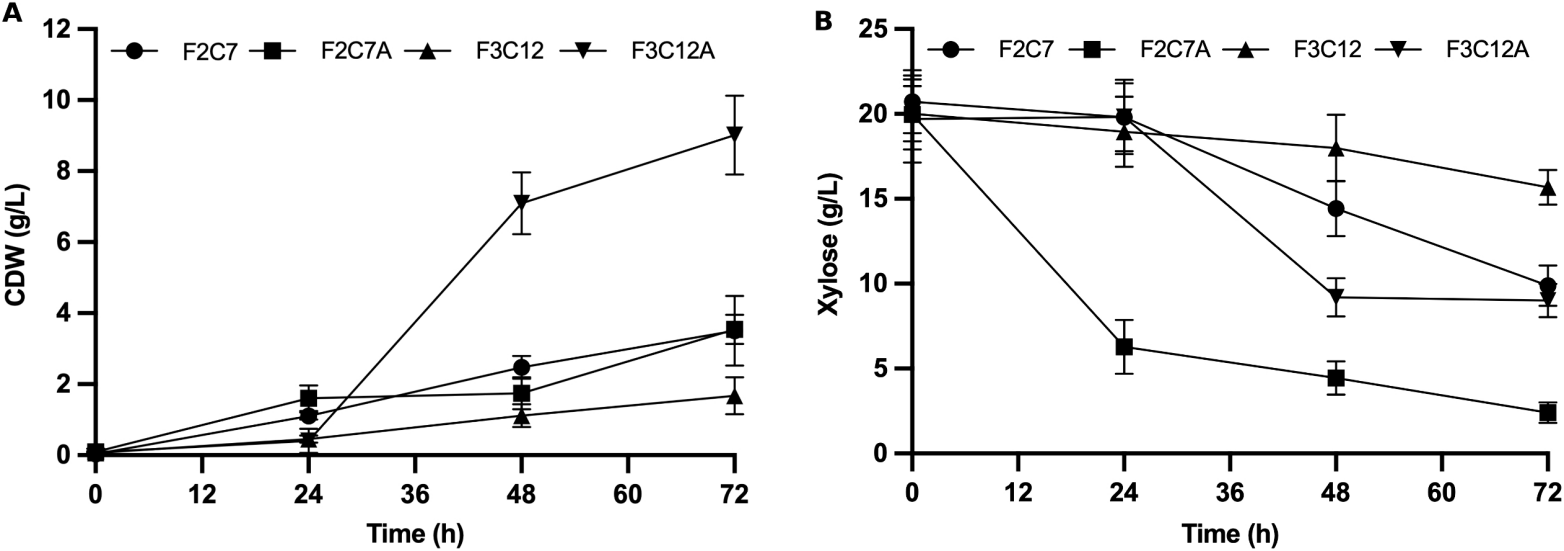
Improved xylose consumption in evolved hybrid strains following adaptive laboratory evolution. (A) Growth curve of hybrid (F2C7 and F3C12) and evolved (F2C7A and F3C12A) strains cultivated in YNB medium containing 20 g/L xylose for 72 h. (B) Xylose consumption kinetics of hybrid and evolved strains. Data represent mean ± SD of three independent biological replicates.

To explore the transcriptional adaptations of the evolved strain F2C7A to different carbon sources, RNA-seq analysis was performed after 24 h of aerobic cultivation in media containing xylose (XIL), glucose (GLU), or both sugars (XILGLU). Differential gene expression was evaluated through three pairwise comparisons: XIL vs GLU, XILGLU vs GLU, and XILGLU vs XIL. The largest transcriptomic shift was observed in the comparison between xylose and glucose-grown cells (XYL vs GLU), which revealed 1,128 differentially expressed genes (DEGs), including 515 upregulated and 613 downregulated genes (Fig 5A-C). This extensive transcriptional response reflects the broad metabolic reprogramming required for xylose assimilation compared with glucose metabolism. In contrast, the mixed substrate versus xylose comparison (XILGLU vs XIL), identified 1,042 DEGs (579 upregulated, 463 downregulated), indicating that glucose exerts a strong regulatory effect even in the presence of xylose. The transcriptional response to the mixed substrate compared to glucose alone (XILGLU vs GLU) was minimal, with only 17 DEGs detected (14 upregulated, 3 downregulated), suggesting that glucose dominates the regulatory response and suppresses the transcriptional activation typically required for xylose metabolism (Fig 5A, B).

Functional enrichment analysis of DEGs (S1 Table) revealed that cells grown on xylose exhibited increased expression of genes related to carbon and fatty acid metabolism, oxidative phosphorylation, and the pentose phosphate pathway, consistent with respiratory catabolism. In contrast, cells grown on glucose showed enrichment in genes involved in ribosome biogenesis, purine metabolism, and amino acid biosynthesis—hallmarks of fermentative, rapid-growth metabolism.

### Transcription factors

Changes in gene expression regulation have shown significant impact on the phenotypic evolution of yeast strains. Potential transcription factors (TFs) regulating the differentially expressed genes were identified using the YEASTRACT database. In the comparison between xylose and glucose conditions (XIL vs. GLU), a total of 17 TFs were upregulated and 9 were downregulated (S1 Fig). Several enriched TFs (*PPR1*, *GCN4*, *RME1*, *DAL80*, and *STB4*) are associated with nutrient stress responses and biosynthetic limitations [26], which are consistent with the conditions observed during growth on xylose. The upregulation of *GCN4* and *DAL80* in the presence of xylose suggests a potential link between nitrogen metabolism and the cellular metabolic response to xylose assimilation, particularly considering that Dal80p regulates genes involved in glutamine, proline, and urea metabolism [27].

In the comparison between co-fermentation (XILGLU) and xylose alone (XIL), 32 transcription factors were differentially expressed (S1B Fig). Under these conditions, the cells exhibited a higher physiological demand, which may explain the upregulation of *GCR1*, a TF known to activate genes involved in glycolysis [28]. In addition, *RPN4* involved in stress response and proteasome regulation and associated with ethanol-induced stress responses [29] was also upregulated.

### Sugar transporters

In F2C7A, xylose uptake was associated with the overexpression of several nonspecific sugar transporters. Notably, *STL1*, a glycerol/H⁺ symporter typically repressed by glucose and induced under stress conditions or the late stages of fermentation to help maintain cellular redox balance [30], was strongly upregulated (Log₂FC = 3.62). Although *STL1* is not canonically described as a xylose transporter, its induction under these conditions suggests a broader adaptive function in facilitating sugar uptake during metabolic stress. In other yeasts, such as *Kluyveromyces marxianus*, homologs of *Sc_STL1* have been identified as a low-affinity xylose transporter in addition to their role in glycerol transport [31]. Interestingly, the gene annotated as *HGT1* (also known as *OPT1*, YJL212C), which encodes a glutathione/oligopeptide transporter, was significantly overexpressed (Log₂FC = 4.14) in xylose-grown cells. Although *OPT1/HGT1* is not a sugar transporter, its induction may reflect a cellular response to oxidative stress or altered redox homeostasis during xylose metabolism. Additionally, *HXT8*, a low-affinity hexose transporter expressed under glucose-limited conditions [32], was upregulated (Log₂FC = 2.79), suggesting that the evolved strain activates multiple transport systems to optimize carbon utilization in xylose medium.

The maltose transporters *MAL11* and *MAL61* were also upregulated in xylose media, suggesting potential involvement in alternative sugar uptake. *SNF3* expression was slightly higher in xylose, indicating a possible regulatory role in low-glucose environments, while *RGT2* levels remained relatively unchanged.

### Expression changes in central metabolism under different carbon sources

**Fig 6:**
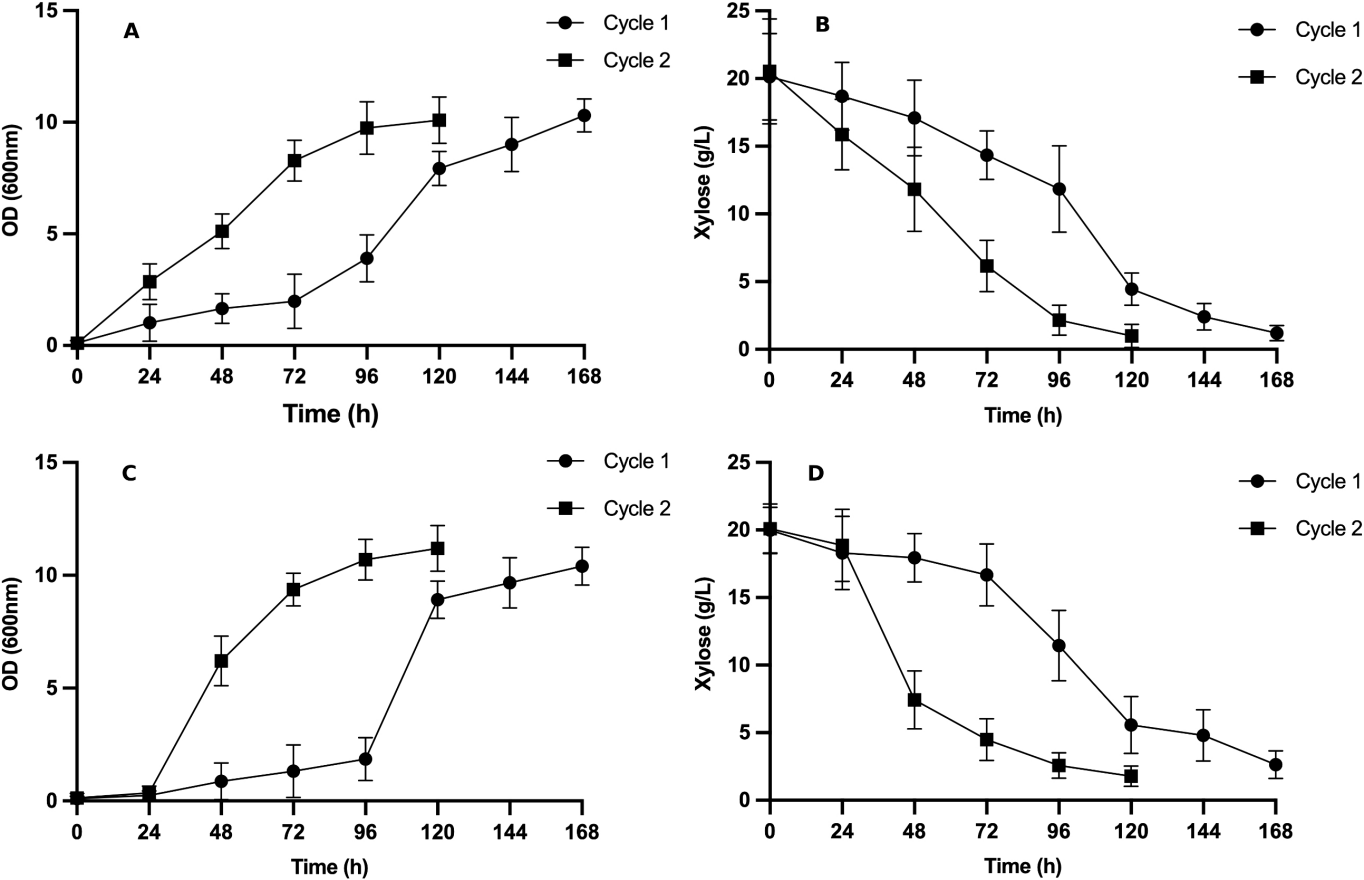
Adaptive laboratory evolution improves growth kinetics and xylose consumption in hybrid yeast strains. Growth kinetics of hybrid strains F2C7 (A) and F3C12 (C) during ALE cycles in synthetic medium containing 20 g/L xylose. Xylose consumption profiles of F2C7 (B) and F3C12 (D). From these adapted populations, isolates F2C7A and F3C12A were subsequently obtained and used in further experiments. Data represents the mean ± SD of three independent biological replicates.

Genes encoding enzymes of the glycolytic pathway and pentose phosphate pathway (PPP) did not show significant differential expression across the conditions tested (Fig 6). However, *GPD1* and *GPP1,* genes involved in glycerol biosynthesis, were upregulated in the presence of glucose, indicating an active fermentative metabolism. Under xylose-only conditions, a clear shift toward respiratory metabolism was observed. Genes involved in the TCA cycle and oxidative phosphorylation such as *ACO1*, *SDH1*, and *FUM1*, were significantly upregulated, consistent with the high biomass yield and low ethanol production in this condition. Genes related to acetate metabolism, *ALD2* and *ADH6*, were also overexpressed under xylose, suggesting redox balancing through alternative pathways.

The oxidoreductive pathway for xylose assimilation was strongly activated in xylose-containing media. *XYL2* displayed the highest induction (Log_2_FC = 6.4) in xylose-grown cells, while *XYL1* and *YJR096W*, encoding putative xylose reductases, were also upregulated in all xylose-containing media. Interestingly, *XKS1*, which encodes xylulokinase and directs xylulose into the PPP, showed only moderate upregulation. Furthermore, the limited expression of *TAL1* and *TKT1*, enzymes from the non-oxidative branch of the PPP, suggests a regulatory bottleneck that may contribute to xylitol accumulation observed under xylose-only conditions.

In the mixed-sugar condition (XILGLU), gene expression patterns reflected a hybrid metabolic state. *TDH1*, *PGK1*, and *ENO2*, showed intermediate expression levels, while mitochondrial genes remained upregulated, suggesting a respirofermentative profile, possibly due to sequential sugar utilization.

Schematic representation of glycolysis, the pentose phosphate pathway, and the tricarboxylic acid (TCA) cycle showing log₂ fold change (Log₂FC) values for differentially expressed genes. Comparisons include xylose vs glucose (XIL vs GLU), mixed sugars vs xylose (XILGLU vs XIL), and mixed sugars vs glucose (XILGLU vs GLU). Red indicates upregulation, green indicates downregulation, and grey indicates no significant change. Significance was determined using the criteria |log₂FC| ≥ 1 and adjusted *p* < 0.05.

### Nitrogen metabolism

GO and KEGG enrichment analyses indicated that protein metabolic processes (GO:0019538) and ribosomal structural genes (GO:0003735) were underrepresented in xylose-grown cells. The transcription factor *GCN4*, a key activator of amino acid biosynthesis under nutrient limitation, was upregulated in the XIL condition (Log₂FC = 1.2), whereas *FHL1*, a regulator of ribosomal protein gene expression, was repressed.

Amino acid transporter expression was largely stable between conditions, except for *AGP1* (arginine and glutamine transporter), which was upregulated (Log₂FC = 1.1), and *PUT4* (proline transporter), which was slightly downregulated (Log₂FC = –1.3) in xylose.

**Fig 7:**
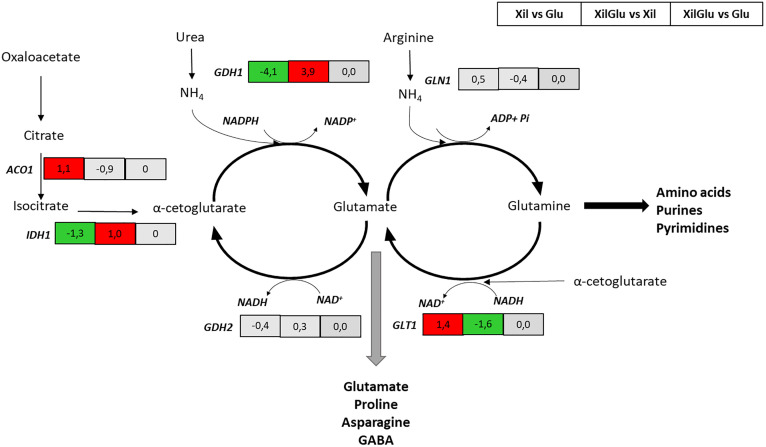
Differential expression of nitrogen metabolism genes in evolved strain F2C7A under mixed-sugar and single-sugar conditions. Schematic representation of nitrogen metabolic pathways showing log₂ fold change (Log₂FC) values for differentially expressed genes. Comparisons include xylose vs glucose (XIL vs GLU), mixed sugars vs xylose (XILGLU vs XIL), and mixed sugars vs glucose (XILGLU vs GLU). Red indicates upregulation, green indicates downregulation, and grey indicates no significant change. Significance was determined using the criteria |log₂FC| ≥ 1 and adjusted *p* < 0.05.

At the interface of carbon and nitrogen metabolism, *IDH1*, encoding NAD ⁺ -dependent isocitrate dehydrogenase, showed mild repression, suggesting reduced α-ketoglutarate production. The NADPH-dependent *GDH1* was strongly downregulated, indicating suppression of ammonium assimilation through this pathway. In contrast, *GLT1* was upregulated, consistent with activation of an alternative glutamate synthesis route from glutamine (Fig 7). Furthermore, *ALT1*, involved in transamination between alanine and glutamate, was upregulated (Log₂FC = 1.5).

## 4. Discussion

Genome shuffling accelerates the improvement of complex traits in yeast by combining beneficial alleles from multiple parental backgrounds through recursive protoplast fusion. Hybrid lines generally undergo progressive genome stabilization involving large-scale chromosomal rearrangements such as aneuploidy, translocations, and partial chromosome loss [33]. The success of hybrid breeding depends on the genetic distance between parental strains; moderate divergence promotes “hybrid vigor” effect, whereas excessive distance can compromise viability through antirecombination and negative epistasis [34,35]. Interspecific *S. cerevisiae* hybrids exhibit variable genomic rearrangements depending on the parental species and stress conditions [36]. Moreover, intergeneric hybrids between *S. cerevisiae* and *S. stipitis* have shown faster glucose utilization and greater xylose consumption than their parents during lignocellulosic hydrolysate fermentation [37]. For instance, the SP2–18 hybrid strain consumed 34% of xylose and reached a final ethanol concentration of 74.65 g/L, outperforming its *S. cerevisiae* parent [11], reinforcing that moderate phylogenetic divergence between species can yield stable, metabolically versatile hybrids suitable for industrial applications.

Interestingly, even the osmotolerant *S. cerevisiae* strain GF16, which is non-recombinant, exhibited measurable xylose consumption after UV mutagenesis. The mutant GFUV06 consumed approximately 18% of the available xylose, suggesting activation of cryptic endogenous pathways for pentose assimilation. The *S. cerevisiae* genome encodes several native xylose-metabolizing genes, including five putative xylose reductases (*GCY1, GRE3, YDL124W, YJR096W,* and *YPR1*), three dehydrogenases (*SOR1, SOR2,* and *XYL2*), and *XKS1* encoding xylulokinase [38]. Although these enzymes exhibit five- to tenfold lower specific activities than those from naturally xylose-fermenting yeasts, random mutagenesis may enhance their expression or cofactor efficiency, uncovering latent metabolic potential for xylose utilization in *S. cerevisiae*.

Developing yeast strains with enhanced xylose metabolism remains a central challenge in second-generation bioethanol production. Here, we combined UV mutagenesis, genome shuffling, and adaptive laboratory evolution (ALE) to develop a hybrid *S. cerevisiae* strain capable of co-fermenting glucose and xylose under aerobic conditions. F2C7A displayed improved sugar uptake, biomass formation, and metabolic flexibility, particularly during growth on xylose.

ALE has been extensively used to improve ethanol tolerance, oxidative stress resistance, and substrate utilization in *S. cerevisiae* [39–41]. In contrast to the evolved strain reported by Xie et al. (2020), which exhibited an 8.3-fold increase in xylose uptake rate but showed no improvement in ethanol yield and accumulated higher levels of xylitol (0.21 g/g) [14], F2C7A demonstrated a more balanced and efficient metabolic response. Although the increase in xylose uptake in F2C7A was more modest (1.8-fold compared with its parental strain), this evolved hybrid achieved a 2.5-fold higher ethanol yield under mixed-sugar conditions and maintained minimal xylitol accumulation. These findings suggest that adaptive evolution in F2C7A favored redox homeostasis and metabolic efficiency rather than excessive flux through the oxidoreductive pathway, leading to improved ethanol productivity without compromising metabolic balance. Under oxygen-limited conditions, F2C7A maintained xylose consumption despite reduced biomass yield, suggesting a metabolic shift toward fermentation. F3C12A prioritized biomass accumulation under aerobic conditions. These divergent outcomes underscore how distinct evolutionary trajectories can shape phenotypic adaptations and highlight the potential of ALE to improve xylose utilization. However, enhancements in ethanol productivity may still require additional metabolic engineering strategies [42,43]. Although the number of ALE cycles applied in this study was limited, the rapid emergence of adaptive phenotypes suggests that prior UV mutagenesis and genome shuffling generated sufficient genetic diversity to accelerate selection. Similar short-term ALE strategies have been reported to yield stable evolutionary gains in industrial yeasts with high genomic plasticity [44].

Fermentation assays revealed clear metabolic shifts depending on the carbon source. As expected, *S. cerevisiae* exhibited the Crabtree effect in glucose media, favoring fermentation even under aerobic conditions, while xylose induced a predominantly respiratory phenotype. Since xylose does not repress respiration to the same extent as glucose [45], its limited uptake likely prevented full activation of glucose-like repression, explaining the low ethanol yield observed. The upregulation of respiratory pathways in F2C7A under xylose therefore appears to respond to increased ATP and NAD⁺ demand rather than classical carbon catabolite repression. Importantly, transcriptional evaluation of HKX2 and GCN4 supports the regulatory basis of this shift. HXK2, a key component of the Snf1p/Hxk2p/Mig1p pathway, mediates glucose repression, while Gcn4p has been shown to modulate metabolic flux redistribution linked to the Crabtree effect through Ras/PKA-dependent activation, even when its transcript levels are reduced [46]. The moderate upregulation of GCN4 observed in F2C7A under xylose therefore contribute to maintaining redox balance and amino acid biosynthesis under respiratory conditions. In mixed-sugar medium, F2C7A exhibited a respirofermentative metabolism, with high biomass and ethanol yields. These findings are consistent with previous reports indicating that simultaneous glucose and xylose utilization can positively affect redox homeostasis and maintain high glycolytic flux [47,48]. The observed reduction in xylitol accumulation during co-fermentation suggests that glucose contributes to maintaining a more balanced intracellular redox state, thereby enhancing xylose catabolism. The improved performance of F2C7A under these conditions can be partly explained by redox coupling between glucose and xylose metabolism. Under aerobic conditions, the presence of glucose favors NADH reoxidation via the reduction of acetaldehyde to ethanol, complementing the respiratory reoxidation that occurs as a consequence of the Crabtree effect [49]. In naturally xylose-assimilating yeasts, previous studies have reported increased xylitol accumulation under respiratory conditions when cultures were exposed to low glucose and high xylose concentrations, indicating that glucose availability plays a crucial role in modulating intracellular redox balance and regulating flux through the oxidoreductive pathway [50,51].

Fermentation temperature did not directly affect ethanol yield or xylitol accumulation. Nonetheless, the ability to perform efficiently at 35°C is advantageous for simultaneous saccharification and fermentation (SSF) conditions, which benefits from reduced contamination risk, lower cooling costs, and greater applicability in tropical countries [52]. Moreover, Adaptation to higher temperatures can enhance the fermentative performance of evolved strains, as reported for yeasts able to ferment lignocellulosic hydrolysates at 37–40 °C [53,54].

Transcriptomic analysis revealed global metabolic reprogramming consistent with the physiological changes observed in F2C7A. Under xylose conditions, upregulation of TCA cycle and oxidative phosphorylation genes (e.g., *ACO1*, *FUM1*, *SDH1*) with concurrent downregulation of glycolytic genes (*PGK1*, *TDH1*, *ENO2*) indicated a shift toward respiration, matching the observed higher biomass and lower ethanol yield. Induction of oxidoreductive pathway genes (*XYL1*, *XYL2*, *YJR096W*) further supported this phenotype, while moderate expression of *XKS1* likely prevented ATP depletion and substrate-accelerated cell death under high D-xylulose concentrations [55]. The limited induction of non-oxidative PPP genes (*TAL1*, *TKT1*) suggests a potential bottleneck in the incorporation of xylulose-5-phosphate into central metabolism. Prior studies have reported that duplications in *TAL1*, *RPE1*, and *TKL1* contribute to improved xylose fermentation [56]. Thus, transcriptional upregulation of these genes could further enhance ethanol yield and reduce xylitol accumulation in F2C7A.

Adaptive transcriptional responses were also evident in sugar transport. In *S. cerevisiae*, sugar uptake occurs exclusively through facilitated diffusion, primarily mediated by members of the Hxt transporter family (Hxt1–17p, Gal2p, Snf3p, and Rgt2p). Although these transporters specialize in glucose, some can also mediate xylose uptake, albeit with very low affinity. [57]. In F2C7A, *HXT2* and *HXT8* were upregulated under xylose, suggesting partial activation of the Snf3/Rgt2 signaling pathway and enhanced pentose uptake. Hxt2p is a moderate-affinity glucose transporter that has been shown to support aerobic growth on low xylose concentrations [58]. The role of Hxt8p remains poorly characterized, but its increased expression may indicate adaptive regulation or functional diversification during evolution, possibly enhancing transporter cooperativity. Previous studies have demonstrated that chimeric Hxt transporters can significantly improve xylose transport efficiency in *S. cerevisiae* [59].

Additionally, *STL1* (Log₂FC = 3.6) and *HGT1* (Log₂FC = 4.1) were highly expressed in xylose. While *STL1* is traditionally known as a glycerol/H⁺ symporter repressed by glucose and induced under osmotic or carbon stress, its strong induction may reflect a broader adaptive role in maintaining redox balance and facilitating sugar uptake under carbon-limited conditions. Interestingly, orthologs of *STL1* in *K. marxianus* have been reported to transport xylose with low affinity, further suggesting a possible auxiliary function in pentose uptake.

At the regulatory level, multiple transcription factors (TFs) were differentially expressed. Xylose-grown cells exhibited upregulation of stress-related TFs, including *GCN4*, a central regulator of amino acid biosynthesis activated under nutrient limitation [60]. Gcn4p integrates metabolic signals to coordinate the expression of genes involved in amino acid, carbon, and redox metabolism [61]. Additionally, *STB4*, a less-characterized TF associated with transcriptional reprogramming during nutrient-limited or slow-growth conditions, was also induced under xylose [62].

Transcriptomic data also revealed a regulatory interplay between carbon and nitrogen metabolism. Specifically, the upregulation of *GCN4* and *GLT1*, together with the downregulation of *FHL1* and *GDH1*, suggests a metabolic adaptation aimed at conserving NADPH for xylose reduction and maintaining redox balance. This regulatory shift correlates with the reduced xylitol accumulation and increased ethanol yield observed under mixed-sugar conditions. Upregulation of *GLT1* and repression of *GDH1* indicate a metabolic rerouting that limits NADPH consumption during ammonium assimilation, maintaining a NADPH pool available for the reduction of xylose to xylitol by XR. Concurrently, increased *GDH2* activity, which depends on NAD ⁺ , is likely to enhance NADH regeneration for XDH-mediated xylitol oxidation. This coordinated regulation promotes more efficient redox cycling between NADPH and NADH pools, minimizing xylitol accumulation and improving ethanol formation during mixed-sugar fermentation. Previous studies have shown that *GLT1* overexpression and *GDH1* deletion xylose metabolism by decreasing NADPH demand [63], while *GDH2* overexpression in the absence of *GDH1* can shift XR cofactor preference from NADPH to NADH [64]. Altogether, these results indicate that the transcriptomic reprogramming observed in F2C7A underlies its improved xylose consumption, lower xylitol accumulation, and increased ethanol yield, through coordinated regulation of carbon, nitrogen, and redox pathways.

From an industrial perspective, the traits acquired by F2C7A— enhanced xylose co-utilization, improved redox balance, and tolerance to elevated temperature—have promising implications for large-scale bioethanol production. These characteristics may enhance robustness during variable industrial conditions. Although inhibitor tolerance was not directly assessed in this study, the improved redox homeostasis observed in F2C7A could contribute to increase against common lignocellulosic hydrolysate inhibitors, including furfural, HMF, and acetic acid. Overall, these features position F2C7A as a suitable non-GMO chassis for future strain improvement and process optimization in industrial-scale lignocellulosic bioethanol production.

Despite these improvements, some limitations remain. Xylose utilization in F2C7A reached approximately 33% in mixed-sugar medium, and ethanol yields, although higher than in the parental strain, were moderate compared to fully fermentative recombinant strains. These limitations likely stem from residual constraints in xylose transport efficiency, cofactor recycling between XR and XDH, and incomplete integration of xylose metabolism into central carbon flux. Therefore, while the adaptive strategies applied here significantly improved co-fermentation performance, further optimization through targeted redox cofactor engineering, enhanced non-oxidative PPP activity, or combined evolutionary and metabolic engineering approaches may be necessary to achieve full xylose assimilation and higher ethanol productivity.

While our data provide valuable insights into the transcriptional and physiological adaptations of F2C7A, further studies are necessary to quantify intracellular redox cofactors and to characterize the kinetic properties of the identified transporters.

## Conclusion

This study demonstrates that the sequential application of UV mutagenesis, genome shuffling, and adaptive laboratory evolution (ALE) constitutes an effective non-recombinant strategy to improve the fermentative performance of *Saccharomyces cerevisiae* on mixed glucose–xylose substrates. The evolved hybrid strain F2C7A showed enhanced xylose utilization, reduced xylitol accumulation, and higher ethanol yield under both aerobic and oxygen-limited conditions, indicating an optimized intracellular redox balance between XR and XDH activities. Transcriptomic analyses revealed coordinated regulation of carbon, nitrogen, and redox metabolism—particularly involving *GLT1*, *GDH1*, and *GDH2*—that collectively supported more efficient cofactor recycling and metabolic flexibility.

While xylose assimilation remained incomplete and ethanol yield moderate, these findings underscore the potential of integrating classical mutagenesis and evolutionary engineering to generate robust, non-GMO yeast strains suitable for industrial bioethanol production. Future work should focus on improving xylose transport efficiency, strengthening the non-oxidative pentose phosphate pathway, and assessing tolerance to lignocellulosic inhibitors under scaled-up fermentation conditions.

## Supporting information

S1 Fig Differential expression of transcription factors in evolved strain F2C7A under different carbon source conditions. Bar plots show Log₂ fold change (Log₂FC) values of differentially expressed transcription factors identified in RNA-seq analysis. (A) TFs with significant expression changes in xylose-only medium compared to glucose. (B) TFs with significant expression changes in mixed glucose-xylose medium compared to glucose alone. Positive Log₂FC values indicate overexpression, and negative values indicate repression relative to the glucose condition. Only TFs with |Log₂FC| ≥ 1 and adjusted *p* < 0.05 are shown.
(DOCX)

S1 Table Quality statistics of clean sequencing data. (DOCX)

S2 Table KEGG pathway enrichment analysis of DEGs across all pairwise comparisons. This table includes significantly enriched pathways (FDR-adjusted p-value < 0,05) identified in each contrast (XIL vs GLU and XILGLU vs XIL).
(DOCX)
