# Supplementary material for: Adaptive laboratory evolution and transcriptomic profiling reveal carbon–nitrogen metabolic reprogramming enabling aerobic co-fermentation of glucose and xylose in Saccharomyces cerevisiae
Source: PLoS One. 2026 Jan 30;21(1):e0341927. doi: 10.1371/journal.pone.0341927 (PMC12857955; doi:10.1371/journal.pone.0341927)
Supplement: S1 Fig — Bar plots show Log₂ fold change (Log₂FC) values of differentially expressed transcription factors identified in RNA-seq analysis. (A) TFs with significant expression changes in xylose-only medium compared to glucose. (B) TFs with significant expression changes in mixed glucose-xylose medium compared to glucose alone. Positive Log₂FC values indicate overexpression, and negative values indicate repression relative to the glucose condition. Only TFs with |Log₂FC| ≥ 1 and adjusted p < 0.05 are shown. (DOCX) [file pone.0341927.s001.docx]

**A**

**B**

**S1 Fig. Differential expression of transcription factors in evolved strain F2C7A under different carbon source conditions.**

Bar plots show Log₂ fold change (Log₂FC) values of differentially expressed transcription factors identified in RNA-seq analysis. (A) TFs with significant expression changes in xylose-only medium compared to glucose. (B) TFs with significant expression changes in mixed glucose-xylose medium compared to glucose alone. Positive Log₂FC values indicate overexpression, and negative values indicate repression relative to the glucose condition. Only TFs with |Log₂FC| ≥ 1 and adjusted *p* < 0.05 are shown.
