# Supplementary material for: Adaptive laboratory evolution and transcriptomic profiling reveal carbon–nitrogen metabolic reprogramming enabling aerobic co-fermentation of glucose and xylose in Saccharomyces cerevisiae
Source: PLoS One. 2026 Jan 30;21(1):e0341927. doi: 10.1371/journal.pone.0341927 (PMC12857955; doi:10.1371/journal.pone.0341927)
Supplement: S1 Table — (DOCX) [file pone.0341927.s002.docx]

**Table S1. Quality statistics of clean sequencing data.**

| **Carbon source** | **Samples ID** | **Total raw reads** | **Total clean reads** | **Q20 (%)** | **Total mapped** |
| --- | --- | --- | --- | --- | --- |
| Xylose | X1 | 44450744 | 43756342 | 97.61 | 42265199(96.59%) |
| Xylose | X2 | 57304388 | 56272500 | 97.84 | 54347531(96.58%) |
| Xylose | X3 | 64184498 | 62894388 | 97.77 | 60847231(96.75%) |
| Glucose | D1 | 43250178 | 42499098 | 97.78 | 40956402(96.37%) |
| Glucose | D2 | 51735212 | 50094690 | 97.84 | 48278572(96.37%) |
| Glucose | D3 | 43239510 | 42405514 | 97.9 | 40879105(96.4%) |
| Xylose/Glucose | XD1 | 50808704 | 49953288 | 97.88 | 48219450(96.53%) |
| Xylose/Glucose | XD2 | 44750642 | 44006122 | 97.93 | 42444878(96.45%) |
| Xylose/Glucose | XD3 | 42852526 | 42098328 | 97.97 | 40689993(96.65%) |

**Note**

The RNA-seq data used in this study have been deposited in the NCBI SAR database with BioProject number PRJNA1308275. The SRA records will be accessible with the following link after the publication of this investigation: http://www.ncbi.nlm.nih.gov/bioproject/1308275.
