# Supplementary material for: Adaptive laboratory evolution and transcriptomic profiling reveal carbon–nitrogen metabolic reprogramming enabling aerobic co-fermentation of glucose and xylose in Saccharomyces cerevisiae
Source: PLoS One. 2026 Jan 30;21(1):e0341927. doi: 10.1371/journal.pone.0341927 (PMC12857955; doi:10.1371/journal.pone.0341927)
Supplement: S2 Table — This table includes significantly enriched pathways (FDR-adjusted p-value < 0,05) identified in each contrast (XIL vs GLU and XILGLU vs XIL). (DOCX) [file pone.0341927.s003.docx]

**Table S2. KEGG pathway enrichment analysis of DEGs across all pairwise comparisons.**

This table includes significantly enriched pathways (FDR-adjusted p-value < 0,05) identified in each contrast (XIL vs GLU and XILGLU vs XIL).

| **Comparison** | **KEGG ID** | **Pathway Name** | **Gene Count** | **Gene Ratio** | **Background Ratio** | **Adjusted p-value** | **Regulation** |
| --- | --- | --- | --- | --- | --- | --- | --- |
| XIL vs GLU | sce00500 | Starch and sucrose metabolism | 24 | 0,041 | 0,018 | 0,0004 | Up |
| XIL vs GLU | sce04138 | Autophagy - yeast | 40 | 0,068 | 0,039 | 0,0009 | Up |
| XIL vs GLU | sce00190 | Oxidative phosphorylation | 37 | 0,063 | 0,036 | 0,0016 | Up |
| XIL vs GLU | sce00010 | Glycolysis / Gluconeogenesis | 25 | 0,043 | 0,023 | 0,0039 | Up |
| XIL vs GLU | sce00040 | Pentose and glucuronate interconversions | 11 | 0,019 | 0,007 | 0,0059 | Up |
| XIL vs GLU | sce04145 | Phagosome | 18 | 0,031 | 0,015 | 0,0074 | Up |
| XIL vs GLU | sce00061 | Fatty acid biosynthesis | 10 | 0,017 | 0,007 | 0,0110 | Up |
| XIL vs GLU | sce04011 | MAPK signaling pathway - yeast | 39 | 0,067 | 0,044 | 0,0141 | Up |
| XIL vs GLU | sce00480 | Glutathione metabolism | 19 | 0,032 | 0,018 | 0,0196 | Up |
| XIL vs GLU | sce00053 | Ascorbate and aldarate metabolism | 9 | 0,015 | 0,006 | 0,0196 | Up |
| XIL vs GLU | sce00071 | Fatty acid degradation | 14 | 0,024 | 0,012 | 0,0231 | Up |
| XIL vs GLU | sce00280 | Valine, leucine and isoleucine degradation | 12 | 0,021 | 0,010 | 0,0273 | Up |
| XIL vs GLU | sce01200 | Carbon metabolism | 44 | 0,075 | 0,053 | 0,0273 | Up |
| XIL vs GLU | sce00052 | Galactose metabolism | 11 | 0,019 | 0,009 | 0,0273 | Up |
| XIL vs GLU | sce00330 | Arginine and proline metabolism | 19 | 0,032 | 0,020 | 0,0439 | Up |
| XIL vs GLU | sce03010 | Ribosome | 67 | 0,100 | 0,049 | 5,78E-12 | Down |
| XIL vs GLU | sce03050 | Proteasome | 29 | 0,043 | 0,019 | 1,23E-06 | Down |
| XIL vs GLU | sce00970 | Aminoacyl-tRNA biosynthesis | 26 | 0,039 | 0,021 | 0,0018 | Down |
| XIL vs GLU | sce00670 | One carbon pool by folate | 12 | 0,018 | 0,007 | 0,0035 | Down |
| XILGLU vs XIL | sce03010 | Ribosome | 77 | 0,098 | 0,048 | 4,03E-16 | Up |
| XILGLU vs XIL | sce03050 | Proteasome | 33 | 0,042 | 0,019 | 1,24E-08 | Up |
| XILGLU vs XIL | sce04141 | Protein processing in endoplasmic reticulum | 49 | 0,062 | 0,044 | 0,0208 | Up |
| XILGLU vs XIL | sce00670 | One carbon pool by folate | 12 | 0,015 | 0,007 | 0,0208 | Up |
| XILGLU vs XIL | sce00970 | Aminoacyl-tRNA biosynthesis | 26 | 0,033 | 0,021 | 0,0226 | Up |
| XILGLU vs XIL | sce00190 | Oxidative phosphorylation | 39 | 0,058 | 0,036 | 0,0082 | Down |
| XILGLU vs XIL | sce00500 | Starch and sucrose metabolism | 23 | 0,034 | 0,018 | 0,0082 | Down |
| XILGLU vs XIL | sce04011 | MAPK signaling pathway - yeast | 45 | 0,067 | 0,044 | 0,0082 | Down |
| XILGLU vs XIL | sce00010 | Glycolysis / Gluconeogenesis | 26 | 0,039 | 0,023 | 0,0118 | Down |
| XILGLU vs XIL | sce04138 | Autophagy - yeast | 40 | 0,060 | 0,039 | 0,0118 | Down |
| XILGLU vs XIL | sce04145 | Phagosome | 19 | 0,028 | 0,015 | 0,0135 | Down |
| XILGLU vs XIL | sce00040 | Pentose and glucuronate interconversions | 11 | 0,016 | 0,007 | 0,0162 | Down |
| XILGLU vs XIL | sce00280 | Valine, leucine and isoleucine degradation | 13 | 0,019 | 0,010 | 0,0396 | Down |
| XILGLU vs XIL | sce04113 | Meiosis - yeast | 43 | 0,064 | 0,046 | 0,0487 | Down |
| XILGLU vs XIL | sce00053 | Ascorbate and aldarate metabolism | 9 | 0,013 | 0,006 | 0,0487 | Down |
| XILGLU vs XIL | sce00410 | β-Alanine metabolism | 14 | 0,021 | 0,012 | 0,0487 | Down |
| XILGLU vs XIL | sce01200 | Carbon metabolism | 48 | 0,072 | 0,053 | 0,0487 | Down |
| XILGLU vs XIL | sce00360 | Phenylalanine metabolism | 11 | 0,016 | 0,008 | 0,0487 | Down |
| XILGLU vs XIL | sce00480 | Glutathione metabolism | 19 | 0,028 | 0,017 | 0,0487 | Down |
